# Dopamine receptor 3 might be an essential molecule in 1-methyl-4-phenyl-1,2,3,6-tetrahydropyridine-induced neurotoxicity

**DOI:** 10.1186/1471-2202-14-76

**Published:** 2013-07-31

**Authors:** Yan Chen, Ying-yin Ni, Jie Liu, Jia-wei Lu, Fang Wang, Xiao-lin Wu, Ming-min Gu, Zhen-yu Lu, Zhu-gang Wang, Zhi-hua Ren

**Affiliations:** 1 Department of Medical Genetics, Shanghai Jiao Tong University School of Medicine, Shanghai 200025, China; 2 Biopharmaceutical R&D Center, Chinese Academy of Medical Sciences & Peking Union Medical College, Suzhou 215126, China; 3 Research Centre for Experimental Medicine, Rui-Jin Hospital Affiliated to Shanghai Jiao Tong University School of Medicine, Shanghai 200025, China; 4 Shanghai Research Centre for Model Organisms, Shanghai 201210, China; 5 Shanghai Institute of Traumatology and Orthopaedics, Rui-Jin Hospital Affiliated to Shanghai Jiao Tong University School of Medicine, Shanghai 200025, China; 6 Institute of Health Sciences, Shanghai Institutes for Biological Sciences, Chinese Academy of Sciences & Shanghai Jiao Tong University School of Medicine, Shanghai 200025, China

**Keywords:** Dopamine receptor 3, 1-Methyl-4-phenyl-1,2,3,6-tetrahydropyridine-induced neurotoxicity, Parkinson’s disease

## Abstract

**Background:**

1-Methyl-4-phenyl-1,2,3,6-tetrahydropyridine (MPTP) induces Parkinson’s disease (PD)-like neurodegeneration of dopaminergic neurons in the substantia nigra pars compacta (SNpc) via its oxidized product, 1-methyl-4-phenylpyridinium (MPP+), which is transported by the dopamine (DA) transporter into DA nerve terminals. DA receptor subtype 3 (D3 receptor) participates in neurotransmitter transport, gene regulation in the DA system, physiological accommodation via G protein-coupled superfamily receptors and other physiological processes in the nervous system. This study investigated the possible correlation between D3 receptors and MPTP-induced neurotoxicity. A series of behavioral experiments and histological analyses were conducted in D3 receptor-deficient mice, using an MPTP-induced model of PD.

**Results:**

After the fourth MPTP injection, wild-type animals that received 15 mg/kg per day displayed significant neurotoxin-related bradykinesia. D3 receptor-deficient mice displayed attenuated MPTP-induced locomotor activity changes. Consistent with the behavioral observations, further neurohistological assessment showed that MPTP-induced neuronal damage in the SNpc was reduced in D3 receptor-deficient mice.

**Conclusions:**

Our study indicates that the D3 receptor might be an essential molecule in MPTP-induced PD and provides a new molecular mechanism for MPTP neurotoxicity.

## Background

MPTP targets the basal ganglia and/or the substantia nigra, inducing most of the biochemical, pathological, and clinical features akin to Parkinson’s disease (PD) in both human and non-human primates [1]. MPTP, which is lipid-soluble, readily penetrates the blood–brain barrier and enters the brain cells. Because it is amphiphilic, it is captured in acidic organelles, mostly lysosomes, of astrocytes [2]. MPTP itself does not appear to be toxic, but its oxidized product, 1-methyl-4-phenylpyridinium (MPP+), is toxic. Astrocytes and serotonergic neurons contain monoamine oxidase B (MAO-B), which converts MPTP to MPP + [1,2]. The toxic oxidation product reaches the extracellular fluid and is transported by the dopamine (DA) transporter into DA nerve terminals [1,2]. Inhibition of either MAO-B or the DA transporter protects against MPTP-generated MPP+toxicity [1-4]. It has been suggested that MPP+toxicity is dependent on a mitochondrial concentrating mechanism via selective uptake [1,2]. Energy-driven mitochondrial uptake of MPP + results in sufficiently high concentrations of the toxin to interfere with mitochondrial respiration [1,2]. Blockade of mitochondrial respiration has two cytotoxic consequences. First, it impairs ATP formation, resulting in the inhibition of energy-dependent processes such as Ca^2+^ transport. This result in an elevation of intracellular Ca^2+^, leading to the activation of Ca^2+^-dependent enzymes and resulting in cellular damage. Second, MPP+appears to support the occurrence of oxidative stress [1,2].

DA receptor agonists are currently useful medications for PD, and are even regarded as a first choice to delay levodopa therapy [5-7]. This pharmacological evidence suggests that DA and DA receptors participate in the genesis of the behavioral and neurochemical Parkinsonian phenotype [8]. On the basis of biochemical, pharmacological, and physiological criteria, DA receptors have been classified into two subfamilies: D1 (which includes D1 and D5 subtypes) and D2 (which includes D2, D3, and D4 subtypes) [8-12]. All DA receptors share three major structural characteristics: (1) seven hydrophobic transmembrane stretches, (2) significant amino acid sequence identity among different subfamilies within these transmembrane regions, and post-translational modifications such as glycosylation and phosphorylation, and (3) conserved amino acid residues that are involved in the interaction with G-proteins and binding agonists. Genes encoding members of the DA receptor family are part of a larger superfamily of genes comprising the G protein-coupled superfamily receptors (GPCRs) [8-12]. Scientists have attempted to clarify the significance of the specific effects of DA receptors on DA-related neuronal physiology and pathophysiology using genetically modified mice. D3 receptor-deficient mice do not exhibit Parkinsonism, while D3 mutant mice exhibit hyperactivity in novel and exploratory environments and increased rearing behavior [13-15]. Administration of cocaine results in increased mRNA levels of c-fos and dynorphin in the dorsal and ventral striatum of D3-receptor knockout mice, indicating that the D3 receptor plays a role in gene regulation in the DA system [16]. Moreover, research has indicated that the constitutive inactivation of D3 receptors leads to a decrease in agonist-promoted D1 receptor activity [17,18]. Different quantitative expression profiles of *parkin*, the causative gene for autosomal recessive juvenile PD, were observed in D3 knockout mice compared with control mice. The parkin protein has an E3 ubiquitin-ligase activity, and loss of parkin function may result in accumulation of unnecessary molecules that lead to the degeneration of neurons. Parkin showed a higher intensity in D3 receptor knockout mice compared with wild-type mice [19].

Although research has been conducted to demonstrate the role of the D3 receptor in PD-related neuropathology, no investigation has been performed to identify the role of the D3 receptor in MPTP-induced nervous lesions and pathology. In this study, we demonstrate that D3 receptor-deficient mice display attenuated MPTP-induced neurotoxicity. Our study contributes to further understanding of MPTP-induced PD and may lead to a new treatment approach for control of this disease.

## Methods

### Mice and MPTP treatment

Littermate D3 receptor-deficient (D3−/−) male mice (Ensemble number: ENSMUSG00000022705) and wild-type mice (provided by Dr. Xu M., University of Chicago), weighing 30–40 g and 2–3 months old were used in this study. The mice were developed by gene-targeting techniques, maintained on a 129/C57 mixed background, as described previously [14], and derived from heterozygous matings. DNA was isolated from mouse tail biopsies, and the D3−/− knockout genotype was confirmed by PCR using the forward primer (NeoF: 5′CATTCTGCACGCTTCAAAAGCG3′) and reverse primer (NeoR: 5′TTTCTCGGCAGGAGCAAGGTG3′). The PCR protocol was as follows: after denaturing at 94°C for 2 min, all reactions were followed by 30 cycles (94°C for 30 s, 60°C for 30 s, and 72°C for 30 s) using the Basic PCR mix (Biovisualab, Shanghai, China). Mice were individually housed on arrival in a stress-minimized specific pathogen-free facility with free access to food and water. After adaptation to a standard 12-h light/dark cycle (lights on from 6:00 am to 6:00 pm) for 3–4 weeks, the animals were randomly assigned to four groups: wild-type mice injected with saline, wild-type mice injected with MPTP, D3−/− mice injected with saline, and D3−/− mice injected with MPTP.

To establish an MPTP-induced acute mouse model for PD, animals received nine intraperitoneal injections of MPTP (5 or 15 mg/kg/day; Sigma, St. Louis, MO) or an equal volume of saline as a control [20]. All animal experiments were conducted in accordance with internationally recognized guidelines for animal experiments (“Animal Research: Reporting In Vivo Experiments” (ARRIVE) guidelines) and were approved by the Animal Ethics Committee of Shanghai Jiao Tong University School of Medicine (reference number 2010–0018).

#### Behavioral assessment

Locomotor activity was assessed by the following four experiments: the open-field test, rotarod test, pole test, and beam test. All animals were trained twice before each recorded test. All experiments were performed between 13:00 and 17:00, and mice were acclimated to the experimental environment for 30 min prior to training or testing.

The open-field test was performed in a plastic case (length×width×height: 30 × 20 × 20 cm) lit by a 40 W lamp. The base of the case was marked into six grids, and all mice were placed in the same corner to start the test. The case was thoroughly cleaned before each test. Each mouse was tested either three or four times during the experiment. Each test lasted 15 min, and locomotion was recorded at the 5-, 10-, and 15-min time points. Each grid over which the mouse stepped was recorded as one horizontal motion, and each time point at which the mouse’s forelegs were uplifted was recorded as one vertical motion.

The rotarod test was used to further quantify the locomotor activity and degree of motor impairment. Mice were trained at several different rod-rotation speeds. For testing, a 60 mm-diameter rotarod was set to rotate at 10 rpm. The duration from when the mouse was put on the rotarod until the mouse dropped off was recorded; the upper time limit was 180 s. All participants were tested three times, at 30-min intervals.

The pole test was performed according to the method established by Ogawa et al. [21]. Briefly, animals were positioned with their head upward near the top of a rough-surfaced iron pole (1 cm in diameter and 50 cm high). The time taken until they turned completely downward (defined as a “T-turn”) and arrived on the floor was recorded. The maximum time allowed was 120 s.

The challenging beam test was performed according to previous studies using Parkinsonian genetic mouse models. A 2 cm-wide and 50 cm-long beam was set up in a bright laboratory. The mice were placed onto a hanging terminal, and the time required for the mice to traverse through the beam to a platform terminal was recorded.

#### Histology and immunohistochemistry

At the end of the behavioral testing period, brain segments were subjected to histopathologic analysis. The number of damaged neurons was calculated as the average number of cells counted per field. The general criteria to score damaged cells included hyperchromatic nuclei and cytoplasmic vacuolation. The number of damaged neurons was visually estimated on three sections from each animal for each experimental group. Coronal sections of the SNpc were cut on a vibratome, stained with anti-tyrosine hydroxylase (anti-TH) antibody (polyclonal rabbit anti-TH, 1:1000; Abcam, Cambridge, UK), followed by biotinylated secondary antibody (goat anti-rabbit IgG; Abcam, Cambridge, UK), and avidin-horseradish peroxidase conjugate (ABC, Vector, Peterborough, UK) according to the manufacturer’s instructions. The antibody binding was visualized with 3,3′-diaminobenzidine (Sigma, Milan, Italy) as the chromogen. TH-positive neurons were enumerated on three serial sections per animal. TH-labeled neurons were scored as positive only if their cell bodies included well-defined nuclear counterstaining. An independent pathologist who was blind to the specifics of the experiment determined the number of TH-positive neurons.

#### Statistical analysis

Because the behavioral data obtained in the locomotor activity tests were not normally distributed, comparisons of dose–response effects were conducted with Kruskal–Wallis one-way analysis of variance (ANOVA). Comparisons between the two groups of mice at each dose were conducted with the Mann–Whitney test. The remaining observations were compared with the Mann–Whitney test. *p* < 0.05 was considered statistically significant.

## Results

### D3 receptor-deficient mice and MPTP-treated mice

The D3 receptor-deficient mice genotype was confirmed by PCR. Knockout mice displayed a 300-bp band, and the DNA samples isolated from wild-type mice displayed a 200-bp band using forward primer (D3F: 5′GCTCACCACTAGGTAGTTG3′) and reverse primer (D3R: (5′ACCTCTGAGCCAGATAAGC3′) (data not shown). The D3 receptor-knockout mice appeared to be healthy and had no gross physical abnormalities. The mutant mice were fertile, their litter sizes were normal, and there was no obvious sex bias in their offspring. For all subsequent studies, male D3 mutant mice were used, with male wild-type littermates as controls.

The MPTP-induced neurotoxicity mouse model was established by MPTP dose grouping. After the fourth MPTP injection and in all subsequent experiments, animals that received 15 mg/kg per injection displayed significant bradykinesia based on the open-field test and rotarod test. No significant behavioral change was observed among the mice that received 5 mg/kg MPTP per injection or the mice injected with an equal volume of saline (data not shown). Thus, 15 mg/kg/day was established as the adaptive dose for MPTP-induced neurotoxicity.

### D3 receptor-deficient mice displayed no change in the time taken for the “T-turn” in the pole test

**Figure 1:**
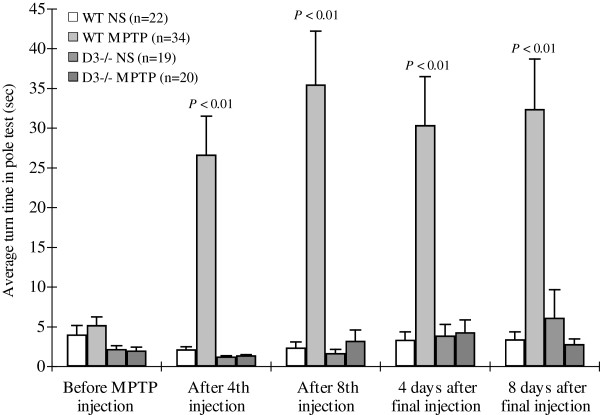
**Time taken for the “T-turn” in the pole test.** To observe behavioral changes in wild-type and D3 receptor-deficient mice treated with MPTP, wild-type mice injected with saline, wild-type mice injected with MPTP, D3 receptor-deficient mice injected with saline, and D3 receptor-deficient mice injected with MPTP were subjected to the pole test. All animals were trained three times before performing the test. The time from when the animals were positioned head-upward near the top of the pole until they turned completely downward was recorded and calculated as the mean±SEM. *P* < 0.05 was considered statistically significant. MPTP, 1-methyl-4-phenyl-1,2,3,6-tetrahydropyridine; NS, normal saline.

The pole test was used to examine behavioral changes in the wild-type and D3 receptor-deficient mice treated with MPTP. After three training sessions, the time taken (mean±SEM) from the head-upward position on the pole until the mouse turned its head completely downward, was recorded in the four cohorts: wild-type injected with saline, wild-type injected with MPTP, D3 receptor-deficient injected with saline, and D3 receptor-deficient injected with MPTP. The pre-injection locomotor activity baseline results were 4.1 ± 1.1, 5.2 ± 1.0, 2.2 ± 0.4, and 2.0 ± 0.4 s, respectively (Figure 1). When wild-type mice were injected with MPTP, the “T-turn” times after the fourth injection, after the eighth injection, 4 days after the final injection and 8 days after the final injection, were 26.7 ± 4.9, 35.5 ± 6.7, 30.4 ± 6.1, and 32.4 ± 6.4 s, respectively. These times were significantly longer than those of the wild-type mice injected with saline (*p* < 0.01 at each time point; Figure 1). These data suggest that MPTP treatment changed the locomotor activity of wild-type mice. Interestingly, when D3 receptor-deficient mice were injected with an equivalent dose of MPTP, the “T-turn” times at the four aforementioned time points were 1.4 ± 0.1, 3.3 ± 1.3, 4.3 ± 1.5, and 2.8 ± 0.7 s, respectively. These times were close to those of the wild-type and D3 receptor-deficient mice injected with saline (Figure 1). The above data suggest that the D3 receptor might weakly participate in PD-related dyskinesia and that D3 receptor-deficient mice are protected from MPTP-induced locomotor activity changes.

### Total time taken in the pole test

**Figure 2:**
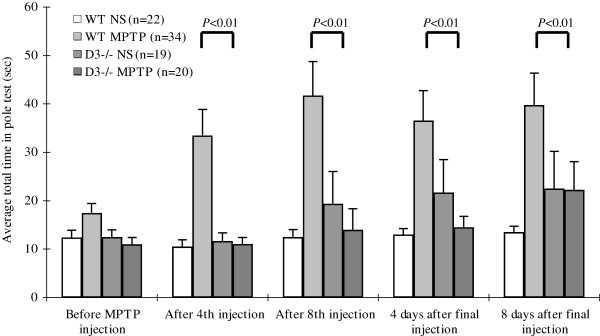
**Total time taken in the pole test.** The total time taken in the pole test by wild-type mice injected with saline, wild-type mice injected with MPTP, D3 receptor-deficient mice injected with saline, and D3 receptor-deficient mice injected with MPTP was recorded as the mean±SEM. *P* < 0.05 was considered statistically significant.

The total time taken in the pole test was defined as the duration from when the animals were positioned head-upward near the top of the pole until they turned completely downward and landed on the floor. The average total times (mean±SEM) of the four cohorts (wild-type injected with saline, wild-type injected with MPTP, D3 receptor-deficient injected with saline, and D3 receptor-deficient injected with MPTP) after training were 12.4 ± 1.5, 17.5 ± 1.9, 12.5 ± 1.5, and 11.0 ± 1.5 s, respectively (Figure 2). When wild-type mice were injected with MPTP, the average total times at the four time points (after the fourth injection, after the eighth injection, 4 days after the final injection and 8 days after the final injection) were 33.5 ± 5.4, 41.8 ± 7.0, 36.6 ± 6.2 and 39.8 ± 6.6 s, respectively. These times were significantly longer than those of the wild-type mice injected with saline (*p* < 0.01 at each time point; Figure 2). When D3 receptor-deficient mice were injected with MPTP, the average total times at the aforementioned four time points were 11.0 ± 1.4, 14.0 ± 4.3, 14.5 ± 2.3 and 22.2 ± 5.9 s, respectively (Figure 2). The average total time taken by the D3 receptor-deficient mice injected with saline was slightly longer than that of wild-type mice at the following three time points: after the eighth injection (19.4 ± 6.7 s), 4 days after the final injection (21.7 ± 6.8) and 8 days after the final injection (22.5 ± 7.7). The *p* value at each time point was < 0.05, which might suggest that the D3 receptor-knockout genotype altered locomotor activity (Figure 2). With the exception of the time point 8 days after the final injection (22.2 ± 5.9 s), the average total time taken by the D3 receptor-deficient mice injected with MPTP was close to that of wild-type mice injected with saline (*p* = 0.04; Figure 2).

### Total time taken in the beam test

**Figure 3:**
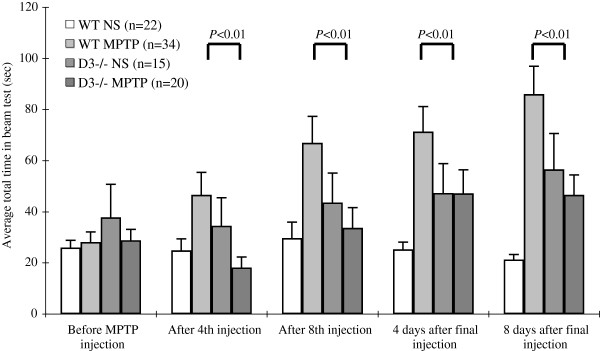
**Average total time taken in the beam test.** The total time taken in the beam test by wild-type mice injected with saline, wild-type mice injected with MPTP, D3 receptor-deficient mice injected with saline, and D3 receptor-deficient mice injected with MPTP was recorded as the mean±SEM. *P* < 0.05 was considered statistically significant.

To further confirm whether the D3 receptor-deficient mice could withstand MPTP-induced neurotoxicity, locomotor activity was assessed by the beam test. After training, the average total times taken (mean±SEM) in the beam test by the four cohorts (wild-type injected with saline, wild-type injected with MPTP, D3 receptor-deficient injected with saline, and D3 receptor-deficient injected with MPTP) were 26.0 ± 2.9, 28.1 ± 4.0, 37.9 ± 12.8 and 28.9 ± 4.2 s, respectively (Figure 3). When wild-type mice were injected with MPTP, the average total times at the four time points (after the fourth injection, after the eighth injection, 4 days after the final injection, and 8 days after the final injection) were 46.7 ± 8.8, 66.9 ± 10.4, 71.4 ± 9.8 and 86.0 ± 10.9 s, respectively. These times were significantly longer than those of the wild-type mice injected with saline (Figure 3). When D3 receptor-deficient mice were injected with MPTP, the average total times at the four aforementioned time points were 18.2 ± 4.1, 33.7 ± 7.9, 47.2 ± 9.3 and 46.6 ± 7.8 s, respectively. These times were significantly shorter than those of wild-type mice injected with saline *(p* < 0.01 at each time point; Figure 3). Similar to the results of the pole test, the average total time taken by the D3 receptor-deficient mice injected with saline was slightly longer than that of wild-type mice at the following two time points: 4 days after the final injection (47.4 ± 11.5 s) and 8 days after the final injection (56.6 ± 14.1 s). Taken together, the above data further suggest that D3 receptor-deficient mice can withstand MPTP-induced locomotor activity changes.

### Neurohistological assessment

**Figure 4:**
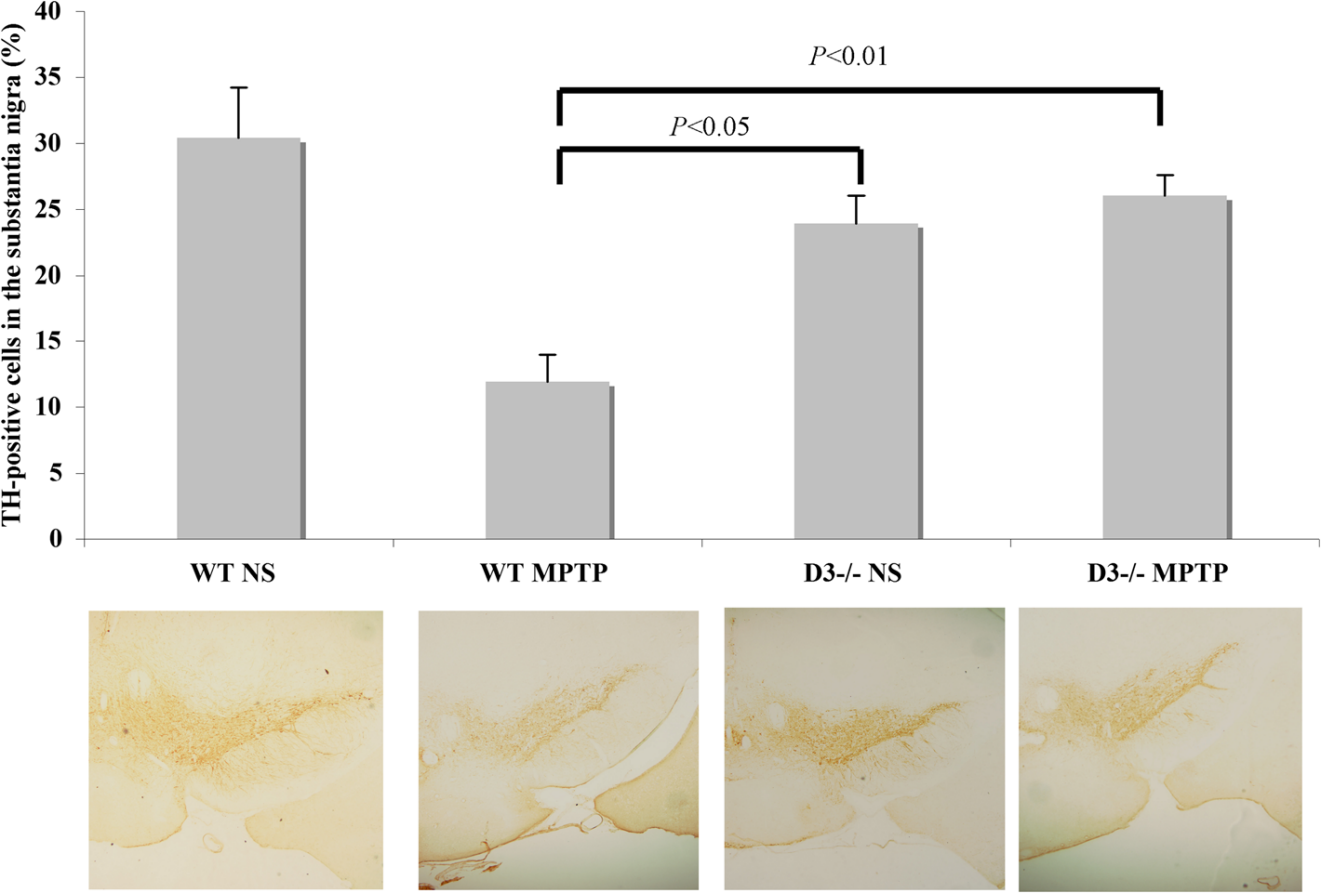
**Neurohistological assessment.** Immunostaining of tyrosine hydroxylase-positive neurons in the SNpc and terminals in the striatum. Eight days after the final MPTP injection, all mice were subject to neurohistological assessment by immunohistochemical staining for tyrosine hydroxylase in the SNpc (bottom). Tyrosine hydroxylase-positive cells were scanned and analyzed by software; results are presented as mean±SEM. *P* < 0.05 was considered statistically significant.

Because MPTP may induce PD-like neurodegeneration of dopaminergic neurons in the SNpc and because the above data show that D3 receptor-deficient mice can withstand MPTP neurotoxicity, we next evaluated the neurohistological changes in the four cohorts. Tyrosine hydroxylase (TH) is the enzyme responsible for catalyzing the conversion of the amino acid L-tyrosine to L-3,4-dihydroxyphenylalanine in the central nervous system. Changes in TH expression are associated with neurodegenerative diseases such as Alzheimer’s disease, PD, and Huntington’s disease. We therefore examined MPTP-induced changes in TH expression. All mice were subjected to neurohistological assessment 8 days after the final injection. Immunohistochemical staining for TH in the SNpc showed that the neuron density and distribution in MPTP-treated wild-type mice were reduced and withered compared with those of the wild-type mice injected with saline. Both D3 receptor-deficient cohorts displayed only slight or no neurological damage (Figure 4, bottom). When these sections were scanned and analyzed by software, the TH-positive cells of the substantia nigra sampled from wild-type mice injected with saline, wild-type mice injected with MPTP, D3 receptor-deficient mice injected with saline, and D3 receptor-deficient mice injected with MPTP comprised 30.3% ± 3.8%, 11.9% ± 3.1%, 23.9% ± 2.1% and 26.0% ± 1.6% of all neurons, respectively (Figure 4, upper). The MPTP-induced neurological damage in D3 receptor-deficient mice was less extensive than it was in wild-type mice, which suggests that D3 receptor deficiency significantly attenuated TH-positive neuron loss in the SNpc.

## Discussion

PD is a heterogeneous neurodegenerative disease typically diagnosed by its cardinal motor symptoms, including bradykinesia, hypokinesia, rigidity, resting tremor and postural instability [6]. The motor manifestations are attributable to the degeneration of dopaminergic neurons within the SNpc, resulting in DA depletion and derangement of neuronal circuits in the basal ganglia target regions of these neurons [8]. In terms of PD etiology, familial PD is caused by mutations in genes, identified by linkage analyses, that are inherited in an autosomal recessive or dominant manner [22]. Sporadic PD is considered to be a complex neurodegenerative disease entity with both genetic susceptibility and environmental factors contributing to the etiopathogenesis [23].

MPTP has been reported to cause chronic Parkinsonism in humans and non-human primates and long-lasting striatal DA depletion in mice [24]. Although there are two major differences between MPTP-induced PD and real PD (lack of Lewy bodies and the fact that the norepinephrine system is relatively well preserved in the model), MPTP-induced acute animal models provide a feasible model for PD study [24]. Multiple signaling pathways may play a crucial role in the degenerative process in MPTP-treated mice. Studies of the mechanisms of and possible neuroprotection against MPTP neurotoxicity have been conducted for many years; the DA transporter and monoamine oxidase are currently the two main subjects of research [1-4]. The DA transporter (also called the DA active transporter, DAT, or SLC6A3) is a membrane-spanning protein that pumps the neurotransmitter DA out of the synapse and back into the cytosol, from which other transporters sequester DA and norepinephrine into vesicles for later storage and release. DA reuptake via DAT provides the primary mechanism by which DA is cleared from synapses. The neurotoxic effect of MPTP has been tested in mice lacking the DA transporter (DAT−/− mice) [1-4]. Striatal tissue DA content and glial fibrillary acidic protein (GFAP) mRNA expression were assessed as markers of MPTP neurotoxicity, and this study showed that the DAT is a mandatory component for expression of MPTP toxicity *in vivo*[4]. Other studies have shown that monoamine oxidase B (MAO-B) inhibitors attenuate MPTP toxicity in mice [25]. MPTP is metabolized into the toxic cation MPP + by MAO-B; thus, toxic effects of acute MPTP poisoning can be mitigated by the administration of monoamine oxidase inhibitors such as selegiline [1-4].

In this study, a 15 mg/kg dose of MPTP induced obvious nervous damage after 4 daily intraperitoneal injections, and this effect was maintained until 8 days after the final injection (ninth dose). Our initial goal was to compare the physiopathologic transformations of D1 and D3 receptor-deficient mice with MPTP-induced motor disorders. The current data show that D3 receptor-deficient mice display no change or only a slight change in performance in the pole test and beam test. In a series of behavioral assessments, we also found that D3 receptor-deficient mice could withstand the effects of MPTP. Further neurohistological study showed that dopaminergic neuron damage was mild in D3 receptor-deficient mice. MPTP-induced neuron damage requires at least two processes: MAO-B-catalyzed MPP + production and MPP + transport. Although we did not perform a detailed study to determine the exact biological and biochemical role of the D3 receptor in MPTP-induced pathology, our data suggest that the D3 receptor may act directly or indirectly in MPP + transport in the central nervous system and thus protect against nerve damage. We infer that the D3 receptor or D3 receptor-related cellular signaling plays a critical role in MPP + −induced neurotoxicity after MMP + transport by DA transporter. Our study suggests a new mechanism of MPTP-related neuropathology.

## Conclusions

Our study indicates that the D3 receptor is an essential molecule in MPTP-induced PD. Our study provides a new molecular principle for MPTP neurotoxicity and will advance our pharmacological knowledge of D3 receptor agonists.

## Abbreviations

DA: Dopamine; MPP+: 1-methyl-4-phenylpyridinium; MPTP: 1-Methyl-4-phenyl-1,2,3,6-tetrahydropyridine; PD: Parkinson’s disease; SNpc: Substantia nigra pars compacta; TH: Tyrosine hydroxylase.

## Competing interests

The authors declare that there are no conflicts of interest.

## Authors’ contributions

YC and ZR conceived and designed the experiments. YN, JL, JL, FW, XW, MG, ZL and ZW performed the experiments and analyzed the data. All authors read and approved the final manuscript.
